# Psychosocial work exposures of the job strain model and cardiovascular mortality in France: results from the STRESSJEM prospective study

**DOI:** 10.5271/sjweh.3902

**Published:** 2020-09-01

**Authors:** Isabelle Niedhammer, Allison Milner, Béatrice Geoffroy-Perez, Thomas Coutrot, Anthony D ­LaMontagne, Jean-François Chastang

**Affiliations:** 1 INSERM, Univ Angers, Univ Rennes, EHESP, Irset (Institut de recherche en santé, environnement et travail) - UMR_S 1085, Epidemiology in Occupational Health and Ergonomics (ESTER) Team, Angers, France; 2 Centre for Health Equity, Melbourne School of Population and Global Health, University of Melbourne, Melbourne, Victoria 3010, Australia; 3 Santé publique France, Saint-Maurice, France; 4 DARES, Ministère du Travail, Paris, France; 5 Institute for Health Transformation, School of Health & Social Development, Deakin University, Geelong, Victoria 3125, Australia

**Keywords:** cardiovascular disease, cumulative exposure, job-exposure matrix, JEM, ischemic heart disease, job stress, stroke

## Abstract

**Objectives:**

The study aims to explore the prospective associations of the psychosocial work exposures of the job strain model with cardiovascular mortality, including mortality for ischemic heart diseases (IHD) and stroke, using various time-varying exposure measures in the French working population of employees.

**Methods:**

The study was based on a cohort of 798 547 men and 697 785 women for which job history data from 1976 to 2002 were linked to mortality data and causes of death from the national death registry. Psychosocial work exposures from the validated job strain model questionnaire were assessed using a job-exposure matrix (JEM). Three time-varying measures of exposure were studied: current, cumulative, and recency-weighted cumulative exposure. Cox proportional hazards models were used to examine the associations between psychosocial work exposures and cardiovascular mortality.

**Results:**

Within the 1976–2002 period, there were 19 264 cardiovascular deaths among men and 6181 among women. Low decision latitude, low social support, job strain, iso-strain, passive job, and high strain were associated with cardiovascular mortality. Most of these associations were also observed for IHD and stroke mortality. The comparison between the different exposure measures suggested that current exposure may be more important than cumulative (or past) exposure. The population fractions of cardiovascular mortality attributable to job strain were 5.64% for men and 6.44% for women.

**Conclusions:**

Psychosocial work exposures of the job strain model may play a role in cardiovascular mortality. The estimated burden of cardiovascular mortality associated with these exposures underlines the need for preventive policies oriented toward the psychosocial work environment.

Psychosocial work factors have been at the heart of occupational health issues in working populations of developed countries for the last decades. They have been found to influence cardiovascular diseases (1–4). While the literature has been extensive on cardiovascular morbidity, there is a lack of research on the associations between psychosocial work factors and cardiovascular mortality.

Among the existing theoretical models, the job strain model has been widely used for the measurement of psychosocial work factors through Karasek’s Job Content Questionnaire (JCQ) (5). This model relies on three factors: psychological demands (intense and complex workload), decision latitude (skill discretion and decision authority), and social support from supervisor and colleagues. These three factors may have adverse effects on health outcomes, and the model also assumes that the combination of these factors may be still more detrimental for health, in particular job strain (combination of high demands and low latitude) and isostrain (combination of job strain and low support).

A recent literature review summarized the results on the associations between psychosocial work factors and coronary heart disease mortality (6). Twelve studies explored the associations between the job strain model factors and cardiovascular mortality (7–18), three of them were based on the same study sample and hence can be considered as one study only (8, 14, 15). Among eight studies exploring decision latitude, five highlighted the influence of low decision latitude (7, 12, 14, 16, 17). Only one study (13) of five found psychological demands as a risk factor. Three studies explored job strain, and two (13, 14) found that job strain was associated with cardiovascular mortality. Of two studies, only one (11) found an association between iso-strain and cardiovascular mortality. Finally, two studies observed unexpected protective effects of high demands (16), low decision authority and low support from coworkers on cardiovascular mortality (9). These mixed findings make it difficult to draw conclusions about the associations between job strain model factors and cardiovascular mortality.

Three of the previous studies used a job-exposure matrix (JEM) for exposure assessment (7, 12, 17). Such a JEM has at least two major advantages in the topic of psychosocial work exposures: it provides exposure assessment in datasets that do not include any measure of exposure (except job title), and it reduces the bias related to self-reported measures, ie, reporting bias. The use of JEM is not a novel approach in the literature. Indeed, the pioneer studies by Alfredsson et al (19) and Johnson et al (20) already constructed and studied JEM for the job strain model exposures in the 1980–1990s. However, the study of cumulative exposure in association with cardiovascular mortality has been very rare for the studies using JEM and in the literature in general.

Consequently, there is a lack of prospective studies based on large national representative samples of the working population of men and women followed up over a long period of time, exposures from the validated JCQ and derived from JEM, and repeated measures of exposure over time. The present study thus attempted to overcome shortcomings of previous studies by using a very large nationally representative sample of the working population of men and women, evaluating exposures via the validated JCQ and a JEM over a long period of time, constructing various time-varying measures of exposures, and conducting long-term follow-up for exposure and outcome.

The objective of the study was to explore the prospective associations between the psychosocial work exposures of the job strain model and cardiovascular mortality. An additional objective was to examine various time-varying measures of exposure as well as subtypes of cardiovascular mortality.

## Methods

The protocol of the STRESSJEM study has been ­presented in a previous publication (21).

### Study sample

Briefly, the study was based on a nationally representative prospective cohort of the working population of employees that combined the national SUMER survey data from DARES (French Ministry of Labor) and the COSMOP program data from Santé publique France. For the studied sample of 1 511 456 individuals, two sources of data were linked: job history from 1976–2002 (INSEE DADS panel data, a random population-based sample, 1/24^th^, of the French national working population of employees, excluding self-employed workers, agricultural workers/employees, employees of some public sectors and of household activities and extra-territorial organizations) and mortality data and causes of death coded according to the 10^th^, 9^th^, or 8^th^ revisions of the ICD over the period 1976–2005 (French national death registry, INSERM-CépiDc, which is the official statistical organization in charge of mortality and causes of death in France). Data included information about job history over the period 1976–2002, in particular for all jobs held: dates of start and end of job, occupation, economic activity of the company, and company size. Exposure estimates for the validated job strain model questionnaire (JCQ) (22, 23) were assessed through a JEM that was constructed and validated for men and women separately using the national SUMER data. The JEM construction was based on a segmentation method with cross-validation that used the three job title variables: occupation, economic activity of the company, and company size. Validity was assessed in two main steps: comparison between self-reported individual versus JEM exposures, and study of the predictive validity of JEM exposures in association with self-reported health. The JEM estimates were then imputed using the same three job title variables for each job held during 1976–2002 in the COSMOP dataset. One of our previous publications described the construction and study of validity of this JEM (24).

### Outcomes

Three outcomes were studied, namely, deaths for all cardiovascular diseases (I00–I99 in ICD-10 or the corresponding ICD-9 and ICD-8 codes); deaths for ischemic heart diseases (IHD) (I20–I25 or the corresponding ICD-9 and ICD-8 codes); and deaths for stroke (I60, I61, I63, I64 in ICD-10 or the corresponding ICD-9 and ICD-8 codes).

### Measures of exposure

We constructed three time-varying exposure measures, using all jobs held during 1976–2002: (i) current exposure at time i; (ii) cumulative exposure until time i, calculated from an average measure at time i using the estimates of exposure and the time spent in all jobs up to and including time i; and (iii) recency-weighted cumulative exposure at time i, calculated from both past and current exposures and the time elapsed since exposure with higher weights assigned to more recent exposures (25). Psychosocial work exposure effects were assumed to persist for ≤5 years after the end of exposure (26) and decrease linearly over a 5-year period to be null after 5 years. We chose this 5-year period in accordance with the study by Amick et al (26) and also because this was a balanced choice between current exposure at time i only and cumulative exposure that assessed exposure over the whole 26-year follow-up period.

The current exposure measures were: (i) the binary exposures for psychological demands, decision latitude, and social support, derived from the JEM scores dichotomized at the median of the distribution for the first job among the total sample of men and women; (ii) the binary variables for job strain (combination of high demands and low latitude) and isostrain (combination of job strain and low support), constructed from the binary variables of demands, latitude, and support (11, 13); (iii) the 4-category variable related to the four quadrants by Karasek (13), from the combination of demands and latitude, that were: high strain (ie, high demands and low latitude), low strain (ie, low demands and high latitude), passive job (ie, low demands and low latitude), and active job (ie, high demands and high latitude). Two alternative reference groups were used: active job and low strain.

The two cumulative exposure measures (cumulative and recency-weighted cumulative exposure) were based on time-weighted scores for demands, latitude, and support as previously defined and dichotomized. Job strain, isostrain, and the 4-quadrant variable were constructed as mentioned earlier.

### Statistical methods

The hazard ratio (HR) of cardiovascular mortality was estimated according to the studied exposures using Cox proportional hazards models. The proportional hazard assumption checked by graphical analysis was not violated (supplementary material, www.sjweh.fi/show_abstract.php?abstract_id=3902, figures S1–3). It can be noticed that the survival curves at older ages, especially for women, were based on a very low number of persons and had large confidence intervals (CI). The studied exposures were time-dependent variables. Age was used as the time scale. Calendar time and four occupational variables related to biomechanical, physical, chemical, and biological exposures were included as adjustment variables. These four occupational variables were assessed by occupational physicians in the SUMER survey (27) and imputed through a JEM whose construction followed the same methodology as the JEM for the job strain model factors (24). They were used as a marker of social position as they displayed strong social gradients (28). We used a model with delayed entry. Individuals entered the cohort on the 1 January 1976 if they already had a job or when they started a first job within the 1976–2002 period.

As we had full information about exposures over the study period, both mortality during time intervals with job and mortality during the total follow-up (ie, including mortality after the end of last job/exposure) were studied.

For the three exposure measures, we used cardiovascular mortality until the end of last job to study mortality during time intervals with job (called “on-the-job” cardiovascular mortality); thus in this analysis, the follow-up ended at the time of death or at the end date of the last job within the 1976–2002 period, or at the end of follow-up (31 December 2002) if still working at this time, whichever came first.

For the two cumulative exposure measures, as delayed effects may be expected, a second analysis was performed in which the follow-up ended at the time of death or 31 December 2002, whichever came first. Compared to on-the-job cardiovascular mortality, the study of cardiovascular mortality until 2002 included cardiovascular deaths that occurred after the end of last job.

Death for causes other than cardiovascular diseases were censored at that time.

Three types of models were performed which included: (i) each exposure separately, (ii) demands, latitude and support simultaneously, (iii) job strain, isostrain, and the 4-quadrant variable separately. In the model that included demands, latitude, and support together, we tested the interaction term between high demands and low latitude, following Karasek’s hypothesis.

Akaike’s Information Criterion (AIC) was used to compare the three models with the three exposure measures – current, cumulative, and recency-weighted cumulative exposure – in association with on-the-job cardiovascular mortality to identify the highest relative quality model.

Finally, the population fractions of cardiovascular mortality attributable (PAF) to job strain and isostrain were calculated for France, with *Pe* being the exposure prevalence, and *HR*, the hazard ratio for mortality associated with exposure:

PAF = Pe(HR-1)/[1+Pe(HR-1)]

*Pe* was assessed by the weighted prevalence of job strain (19.94% among men, 28.70% among women) and iso-strain (12.72% among men, 17.44% among women) using the SUMER survey data. *HR* was provided by the present study. We used simulation-modelling techniques for the CI of PAF (29).

As interaction terms were found to be statistically significant between psychosocial work exposures and gender in the total sample, all analyses were performed for men and women separately and using SAS (SAS Institute, Cary, NC, USA) and R statistical software. Gender-related interactions were presented.

### Sensitivity analyses

To check the robustness of the results, we performed the sensitivity analyses: (i) using scores for the measure of exposure instead of binary variables; (ii) adjusting for occupation in addition to the occupational exposures of biomechanical, physical, chemical and biological nature; (iii) imputing the lowest level of exposure instead of the highest level of exposure in case of multiple job-holder (only 3% of the sample had more than one job at the same time); (iv) studying mortality until 2005 instead of 2002; (v) excluding the first years of follow-up (1976–1978).

## Results

### Description of the study sample

The studied sample included 1 496 332 individuals, 798 547 men and 697 785 women (1%, N=15 214, had missing values for job history). Men and women had a mean age of 28 and 27 years, respectively, at entrance in the cohort. There were 13.6 million and 11.6 million person-years in the follow-up period for men and women, respectively. The mean duration of follow-up was 17 years for both genders (21). Over 1976–2002, there were 19 264 cardiovascular deaths (14.19 cases per 10 000 person-years) among men and 6181 (5.35 per 10 000 person-years) among women, including 2988 cardiovascular deaths (2.89 per 10 000 person-years) among men and 474 (0.56 per 10 000 person-years) among women during time intervals with a job (on-the-job cardiovascular mortality). All details about person-years, number of cases, and cases per 10 000 person-years for cardiovascular, IHD and stroke mortality are provided in supplementary table S1. The description of exposures was presented in our study protocol (21). Women had a higher prevalence of exposure to high psychological demands, low decision latitude, low social support, job strain, and iso-strain than men. Low correlations for demands were found with latitude (0.28) and support (-0.18) and a moderate correlation was observed between latitude and support (0.57).

### Associations between psychosocial work factors and cardiovascular mortality

The results are presented in table 1 for current exposure, table 2 for cumulative exposure, and table 3 for recency-weighted cumulative exposure. The results of these tables were very close and can be summarized as follows. Low latitude and low support were found to be risk factors in almost all models. High demands displayed either no effects or protective effects on cardiovascular mortality. Job strain, iso-strain, passive job, and high strain were found to be risk factors in almost all models. No interaction term between high demands and low latitude was found to be statistically significant in the models exploring current exposure to demands, latitude, and support together. Information about person-years, number of cases, and cases per 10 000 person-years for cardiovascular mortality according to the studied exposures of job strain, isostrain, quadrants is provided in supplementary tables S2–5.

**Table 1:** Associations between current exposure and cardiovascular mortality (CM) among men and women. [HR=hazard ratio; CI=confidence interval.]

	Men (N=798 547)	Women (N=697 785)
	
	HR (95% CI) ^a^ (CM=2988)	HR (95% CI) ^a^ (CM=474)
High psychological demands ^b^	0.89 (0.81–0.98)	1.00 (0.81–1.23)
Low decision latitude ^b^	1.36 (1.25–1.48)	1.67 (1.28–2.18)
Low social support ^b^	1.24 (1.14–1.35)	1.56 (1.26–1.93)
High psychological demands ^c^	0.98 (0.88–1.09)	1.01 (0.80–1.27)
Low decision latitude ^c^	1.43 (1.25–1.64)	1.36 (0.98–1.88)
Low social support ^c^	0.93 (0.81–1.07)	1.37 (1.06–1.78)
Job strain ^b^	1.30 (1.16–1.46)	1.24 (0.97–1.58)
Isostrain ^b^	1.26 (1.11–1.42)	1.24 (0.97–1.58)
Quadrants by Karasek ^b, d^		
Active job (reference)	1	1
Low strain	1.08 (0.94–1.23)	0.76 (0.52–1.13)
Passive job	1.37 (1.22–1.54)	1.56 (1.15–2.13)
High strain	1.44 (1.27–1.64)	1.56 (1.14–2.13)
Quadrants by Karasek ^b, c^		
Active job	0.93 (0.81–1.06)	1.31 (0.88–1.94)
Low strain (reference)	1	1
Passive job	1.28 (1.13–1.44)	2.05 (1.34–3.13)
High strain	1.34 (1.15–1.56)	2.04 (1.33–3.14)

a All models were adjusted for calendar time, biomechanical, physical, chemical and biological exposures and age was used as the time scale.

b Each exposure was studied separately.

c Demands, latitude and support were studied simultaneously, ie, adjusted for each other.

d High strain (high demands and low latitude), low strain (low demands and high latitude), passive job (low demands and low latitude), and active job (high demands and high latitude).

**Table 2:** Associations between cumulative exposure and cardiovascular mortality (CM) among men and women. [HR=hazard ratio; CI=confidence interval.]

Follow-up	Men (N=798 547)		Women (N=697 785)	
	
	On-the-job	Until 31/12/2002	On-the-job	Until 31/12/2002
			
	HR (95% CI) ^a^ (CM=2988)	HR (95% CI) ^a^ (CM=19 264)	HR (95% CI) ^a^ (CM=474)	HR (95% CI) ^a^ (CM=6181)
High psychological demands ^b^	1.00 (0.91–1.10)	0.92 (0.89–0.96)	1.02 (0.82–1.27)	0.88 (0.83–0.93)
Low decision latitude ^b^	1.42 (1.30–1.54)	1.19 (1.16–1.23)	1.54 (1.21–1.95)	1.35 (1.26–1.44)
Low social support ^b^	1.23 (1.13–1.33)	1.09 (1.06–1.13)	1.64 (1.30–2.06)	1.10 (1.03–1.16)
High psychological demands ^c^	1.11 (1.00–1.23)	0.96 (0.92–1.00)	0.99 (0.79–1.25)	0.90 (0.84–0.96)
Low decision latitude ^c^	1.41 (1.28–1.55)	1.18 (1.14–1.22)	1.31 (1.00–1.70)	1.29 (1.20–1.40)
Low social support ^c^	1.07 (0.97–1.17)	1.01 (0.98–1.05)	1.50 (1.16–1.93)	1.05 (0.98–1.12)
Job strain ^b^	1.33 (1.18–1.50)	1.19 (1.13–1.24)	1.26 (1.02–1.56)	1.06 (1.00–1.13)
Isostrain ^b^	1.34 (1.18–1.52)	1.17 (1.11–1.23)	1.30 (1.05–1.61)	1.06 (0.99–1.13)
Quadrants by Karasek ^b, d^				
Active job (reference)	1	1	1	1
Low strain	0.87 (0.77–0.99)	1.10 (1.05–1.15)	0.71 (0.44–1.16)	1.08 (0.97–1.21)
Passive job	1.30 (1.16–1.45)	1.23 (1.18–1.29)	1.43 (1.06–1.91)	1.44 (1.33–1.57)
High strain	1.39 (1.22–1.59)	1.28 (1.22–1.34)	1.47 (1.12–1.91)	1.32 (1.21–1.43)
Quadrants by Karasek ^b,d^				
Active job	1.15 (1.01–1.31)	0.91 (0.87–0.96)	1.41 (0.87–2.29)	0.92 (0.83–1.03)
Low strain (reference)	1	1	1	1
Passive job	1.49 (1.33–1.67)	1.13 (1.08–1.17)	2.01 (1.22–3.31)	1.33 (1.19–1.48)
High strain	1.60 (1.38–1.87)	1.17 (1.10–1.23)	2.06 (1.26–3.36)	1.22 (1.09–1.36)

a All models were adjusted for calendar time, biomechanical, physical, chemical and biological exposures and age was used as the time scale.

b Each exposure was studied separately.

c Demands, latitude and support were studied simultaneously, ie, adjusted for each other.

d High strain (high demands and low latitude), low strain (low demands and high latitude), passive job (low demands and low latitude), and active job (high demands and high latitude).

**Table 3:** Associations between recency-weighted cumulative exposure and cardiovascular mortality (CM) among men and women. [HR=hazard ratio; CI=confidence interval.]

Follow-up	Men (N=798 547)		Women (N=697 785)	
	
	On-the-job	Until 31/12/2002	On-the-job	Until 31/12/2002
			
	HR (95% CI) ^a^ (CM=2988)	HR (95% CI) ^a^ (CM=19 264)	HR (95% CI) ^a^ (CM=474)	HR (95% CI) ^a^ (CM=6 181)
High psychological demands ^b^	0.94 (0.85–1.03)	0.91 (0.86–0.97)	1.12 (0.90–1.38)	0.88 (0.78–1.00)
Low decision latitude ^b^	1.37 (1.26–1.48)	1.24 (1.17–1.30)	1.64 (1.27–2.10)	1.53 (1.32–1.77)
Low social support ^b^	1.30 (1.19–1.41)	1.12 (1.06–1.18)	1.78 (1.43–2.22)	1.23 (1.09–1.38)
High psychological demands ^c^	1.04 (0.94–1.15)	0.96 (0.90–1.02)	1.09 (0.87–1.37)	0.91 (0.80–1.04)
Low decision latitude ^c^	1.28 (1.15–1.42)	1.25 (1.17–1.33)	1.30 (0.97–1.74)	1.43 (1.20–1.69)
Low social support ^c^	1.13 (1.02–1.26)	0.97 (0.91–1.04)	1.59 (1.23–2.05)	1.10 (0.95–1.26)
Job strain ^b^	1.26 (1.12–1.42)	1.26 (1.17–1.36)	1.41 (1.13–1.76)	1.16 (1.01–1.33)
Isostrain ^b^	1.23 (1.08–1.40)	1.19 (1.09–1.28)	1.43 (1.14–1.79)	1.16 (1.01–1.33)
Quadrants by Karasek ^b, d^				
Active job (reference)	1	1	1	1
Low strain	0.96 (0.84–1.09)	1.14 (1.05–1.23)	0.74 (0.48–1.15)	1.12 (0.90–1.39)
Passive job	1.33 (1.19–1.49)	1.28 (1.19–1.38)	1.40 (1.03–1.90)	1.61 (1.35–1.93)
High strain	1.36 (1.19–1.55)	1.39 (1.28–1.50)	1.64 (1.24–2.18)	1.54 (1.29–1.83)
Quadrants by Karasek ^b,d^				
Active job	1.04 (0.92–1.19)	0.88 (0.81–0.95)	1.34 (0.87–2.07)	0.90 (0.72–1.11)
Low strain (reference)	1	1	1	1
Passive job	1.39 (1.24–1.56)	1.13 (1.05–1.21)	1.88 (1.19–2.97)	1.45 (1.15–1.81)
High strain	1.42 (1.22–1.65)	1.22 (1.11–1.34)	2.21 (1.42–3.45)	1.38 (1.10–1.73)

a All models were adjusted for calendar time, biomechanical, physical, chemical and biological exposures and age was used as the time scale.

b Each exposure was studied separately.

c Demands, latitude and support were studied simultaneously, ie, adjusted for each other.

d High strain (high demands and low latitude), low strain (low demands and high latitude), passive job (low demands and low latitude), and active job (high demands and high latitude).

### Gender-related interactions

Statistically significant gender-related interactions were observed suggesting differences in exposure–outcome associations by gender. The effect of low support on on-the-job cardiovascular mortality was stronger among women than among men. The association of job strain and iso-strain with cardiovascular mortality until 2002 was stronger among men than women.

### Study of subtypes of cardiovascular mortality

Information about person-years, number of cases, and cases per 10 000 person-years for IHD and stroke mortality according to the studied exposures of job strain, isostrain, quadrants is provided in supplementary tables S2–5. Most of the associations observed in tables 1–3 for cardiovascular mortality remained statistically significant for IHD and stroke mortality among men whereas only a smaller number of the associations remained significant among women, probably due to a low number of cases (supplementary tables S6–11).

### Comparison between models

Tables 2–3 showed that, although the statistical power was higher, the study of cardiovascular mortality after the end of the last job (until 31/12/2002) reduced the effect size of most HR (dilution of the effects over time) compared to the study of on-the-job cardiovascular mortality (during time intervals with job). AIC calculation showed that the model with the lowest value of AIC (ie, the highest relative quality) was the model with current exposure. However, although the AIC value was lower for current exposure, it was not significantly different from the AIC values from the two models with cumulative exposure or recency-weighted cumulative exposure.

### Sensitivity analyses

The sensitivity analyses found similar results compared to tables 1–3, except including occupation as additional adjustment variable, which reduced the statistical significance of the associations (supplementary tables S12–14).

### Population attributable fractions

Following the AIC results, the population fractions of cardiovascular mortality attributable to current exposure to job strain and iso-strain were calculated. For job strain, the PAF were 5.64% (95% CI 3.09–8.42) among men and 6.44% (95% CI 0–14.3) among women. For iso-strain, the PAF were 3.20% (95% CI 1.38–5.09) among men and 4.02% (95% CI 0–9.20) among women.

## Discussion

### Summary of the results

Low decision latitude, low social support, job strain, iso-strain, passive job, and high strain were found to be associated with cardiovascular mortality for both genders. These results were observed for current, cumulative and recency-weighted cumulative exposure. Similar results were found for IHD and stroke mortality, especially among men. The population fractions of cardiovascular mortality attributable to current exposure to job strain were 5.64% and 6.44% for men and women respectively.

### Comparison with the literature

Our study underlined the effect of low decision latitude on cardiovascular mortality. This result is in agreement with the findings of five studies among men mainly (7, 12, 14, 16, 17) and with a recent review and meta-analysis (6), whose results showed that the only statistically significant psychosocial work factor was low latitude in association with coronary heart disease mortality. In our study, high psychological demands were found to be a non-significant or protective factor. These findings echo some rare previous studies that reported no effect (7, 12, 14) or a protective effect of high demands (16). Only one study reported an association between high demands and cardiovascular mortality (13). Low social support was found to be a risk factor in our study. Three previous studies either did not find any effect of low support (12, 16) or found a protective effect of low support from coworkers (9). Our study is thus the first one to show low social support as a risk factor for cardiovascular mortality.

Job strain was associated with cardiovascular mortality in our study, confirming two previous studies (13, 14). We found iso-strain to be a risk factor, which is in agreement with Johnson et al’s study (11) but not that of Padyab et al (16) who reported no effect of iso-strain. Regarding the study of the four quadrants, our study showed that passive job and high strain were both associated with cardiovascular mortality. Two previous studies explored the four quadrants but did not find any associations (16, 18). Our study is thus the first one to report an association between passive job and cardiovascular mortality.

In addition, our HR estimates were very close in effect size to previous estimates published in the literature for both cardiovascular mortality and morbidity. Indeed, the recent review and meta-analysis on job strain in association with cardiovascular mortality (6) provided a summary HR of 1.3 (95% CI 0.9–1.9), though non-significant, and the last update of a meta-analysis (30) on the association between job strain and cardiovascular morbidity reported a summary relative risk of 1.33 (95% CI 1.19–1.49).

All previous studies explored a measure of exposure at baseline in association with cardiovascular mortality, except Johnson et al’s (12), which used a JEM and explored a cumulative exposure based on job history but limited to the past five occupations held by the subjects for ≥2 years, meaning a potential incomplete job history. In addition, Johnson et al’s study examined the association between retrospective cumulative exposure (before 1977) and mortality over the period 1977–1990, leading to an absence of information about exposure during follow-up. The results of this study underlined the effects of low work control on cardiovascular mortality, but no other association was observed except for the combination of low control and low support. The two other studies using JEM also reported an association between low decision latitude and cardiovascular mortality (7, 17).

A small number of studies calculated population attributable fractions, although most of them did so for cardiovascular morbidity and not mortality (3, 29, 31,32). Two studies (33, 34) estimated the fractions of cardiovascular mortality attributable to occupational exposures, including job strain, shift work, noise, exhaust gases, combustion products, and/or environmental tobacco smoke together. Nurminen et al (34) provided estimates of the fractions of cardiovascular fatalities attributable to job strain in Finland that were 16% for men and 19% for women. We may consider that these estimates were high given that Nurminen et al (34) as well as Järvholm et al (33) provided estimates of the fractions of cardiovascular mortality attributable to all occupational exposures together of 23.0% (women) and 20.1% (men) for acute myocardial infarction (33) and of 18.9% (men) and 9.1% (women) for IHD, and of 14.4% (men) and 6.7% (women) for all diseases of the circulatory system (34). In that sense, the attributable fractions of our study are more in line with the fractions of cardiovascular morbidity attributable to job strain, provided by the studies of Kivimaki et al (3), who found an estimate of 3.4% (95% CI 1.5–5.4), and Niedhammer et al (29), who reported an estimate of 4.46% (95% CI 1.26–7.65). The present study adds to the literature by providing estimates for mortality among men and women separately. The PAF estimated among women (6.44% for job strain and 4.02% for isostrain) were found to be non-significant (as 0 was included in the CI). This was explained by the non-significant HR from table 1 used for the calculations. Given the differences between HR (both in statistical significance and effect size) associated with job strain and iso-strain among women between table 1 and tables 2–3 for on-the-job cardiovascular mortality, these PAF estimates may be considered conservative among women. In addition, these PAF estimates may underestimate the global burden of cardiovascular mortality attributable to psychosocial work exposures as only job strain or iso-strain were considered and not other exposures such as, for example, job insecurity or long working hours.

### Limitations and strengths of the study

The strengths of this study deserve to be mentioned. The studied cohort was a very large and national representative sample of the working population of men and women followed up for a long period of time for both exposure and outcome. As the data were routine, participation, response or selection bias were unlikely. However, a healthy worker effect may not be excluded for the association of current exposure and on-the-job cardiovascular mortality, but this would bias the exposure–outcome associations towards the null hypothesis. As we had complete follow-up, there was no attrition bias. As the data were provided by sources that were not the individuals themselves, no reporting bias for both exposure (assessed using a JEM) and outcome (derived from national registry data) could be suspected. We adjusted for physico-biomechanical-chemical-biological exposures that were used as proxies of social position. These occupational exposures are known to display strong social differences, the lower the social groups, the higher the prevalence of exposure (28). Indeed, the results without adjustment for these exposures showed stronger and more significant results than those presented in tables 1–3, underlying the appropriateness of taking them into account. Exposure was assessed by the validated JCQ, and we explored both main factors and combinations of factors. Thus, job strain, isostrain, and the four quadrants were found to be risk factors, which may be explained by the main effects of low decision latitude and low social support on cardiovascular mortality. This point was confirmed by the absence of interaction between high demands and low latitude. Exposure was defined using various time-varying measures. The comparison between the three exposure measures showed that the most recent (current) exposure may be more important than past (cumulative) exposure, although the results were not significant. This may also suggest that repeated measures of exposure (what we called current exposure at each time i) may be most powerful to capture the effects of exposure on cardiovascular mortality. Mortality was provided by the national death registry. We studied two outcomes (on-the-job and until 31/12/2002 cardiovascular mortality) and the results suggested that the effects of exposure on cardiovascular mortality may decrease after the end of exposure (potential reversibility of the effects). Two subtypes of cardiovascular mortality (IHD and stroke mortality) were studied, which has not been done previously. Finally, sensitivity analyses confirmed the robustness of the findings.

However, the study included limitations. Few adjustment variables were available, thus residual confounding bias was possible. We had no data on baseline cardiovascular morbidity. This may have led to a potential selection bias, as people with cardiovascular morbidity at baseline may have changed to less exposed jobs or left the labor market, but this would bias towards the null hypothesis. This may also have led to the reserve bias (bias away from the null) if people with cardiovascular morbidity at baseline changed to jobs with low decision latitude for example. We may think that our study of cumulative and recency-weighted cumulative exposure might have reduced this bias as these exposure measures took past exposure into account. More information would have been useful to control for major cardiovascular risk factors. Nevertheless, controlling for cardiovascular risk factors in the association between psychosocial work factors and cardiovascular mortality may be considered as an over adjustment as these cardiovascular risk factors may be on the causal pathways between exposures and outcome. Although we controlled for other occupational exposures used as proxies of social position, this adjustment may not be completely satisfactory. Our sensitivity analysis additionally adjusted for occupation, a way to control further for social position. However, as occupation was among the job title variables used to construct the JEM, this may have led to over adjustment. Indeed, as expected, although most of the results were found to be similar, the associations were weaker and less significant. The true measure of the exposure–outcome associations may be in between. Although using a JEM may have the advantage of reducing reporting bias, imprecision in the exposure assessment, non-differential misclassification and bias towards the null hypothesis may be present and may have led to an underestimation of the observed associations. Furthermore, the validity of the JEM was lower for psychological demands and social support, which may contribute to explain discrepancies in the results for psychological demands (24). There was rare missing information about some jobs that was treated using midcensoring. As we only had data for 1976–2002, we were unable to assess complete working life-course exposure. We studied two subtypes of cardiovascular mortality (IHD and stroke), but the study of ischemic and hemorrhagic stroke separately provided non-significant results and did not confirm previous results on morbidity reporting an association of job strain with ischemic stroke but not with hemorrhagic stroke (1). Low statistical power was related to both a substantial proportion of stroke cases not specified as hemorrhage or infarction and consequently a low number of cases for each subtype of stroke. The total effect of all these limitations on our results may be difficult to evaluate as they included both over- and underestimations of the exposure–outcome associations.

### Concluding remarks

Our findings suggested that the psychosocial work exposures of low decision latitude, low social support, job strain, iso-strain, high strain, and passive job showed concerning patterns of association with cardiovascular mortality. The same associations were also found for IHD and stroke especially among men. The PAF observed in our study were substantial and reaffirm the need for preventive policies at the workplace to improve cardiovascular health.

## Supplementary material

Supplementary material
