# Supplementary material for: Psychosocial work exposures of the job strain model and cardiovascular mortality in France: results from the STRESSJEM prospective study
Source: Scand J Work Environ Health. 2020 May 21;46(5):542–51. doi: 10.5271/sjweh.3902 (PMC7737793; doi:10.5271/sjweh.3902)

Psychosocial work exposures of the job strain model and cardiovascular mortality in France: results from the STRESSJEM prospective study [1]

by Isabelle Niedhammer, PhD,[2] Allison Milner, PhD, Béatrice Geoffroy-Perez, MD, Thomas Coutrot, PhD, Anthony D -LaMontagne, ScD,2, Jean-François Chastang, PhD

1. *Supplementary tables S1-14 and figure S1-3*
2. *Correspondence to: Isabelle Niedhammer (ORCID: 0000-0002-8042-8925), INSERM U1085 – IRSET - Equipe ESTER, Faculté de Médecine - Université d'Angers, 28 rue Roger Amsler, CS 74521, 49045 ANGERS* Cedex 01, France. [E-mail: isabelle.niedhammer@inserm.fr]

Table S1. Person-years, number of cases, and cases per 10,000 person-years for all studied outcomes

	Person-years	Cardiovascular mortality		Mortality for ischemic heart diseases		Mortality for stroke	
		Number of cases	Cases per 10,000 person-years	Number of cases	Cases per 10,000 person-years	Number of cases	Cases per 10,000 person-years
**On-the-job mortality**							
Men	10,323,038	2,988	2.89	1,451	1.41	513	0.50
Women	8,389,889	474	0.56	100	0.12	176	0.21
**Mortality until 2002**							
Men	13,578,089	19,264	14.19	7,680	5.66	3,435	2.53
Women	11,561,757	6,181	5.35	1,576	1.36	1,617	1.40

Table S2. Person-years, number of cases, and cases per 10,000 person-years for the study of on-the-job mortality among men

	Person-years	Cardiovascular mortality		Mortality for ischemic heart diseases		Mortality for stroke	
		Number of cases	Cases per 10,000 person-years	Number of cases	Cases per 10,000 person-years	Number of cases	Cases per 10,000 person-years
**CURRENT EXPOSURE**							
**Job strain**							
Exposed	911,945	402	4.41	186	2.04	73	0.80
Non-exposed	6,925,398	2,586	3.73	1,265	1.83	440	0.64
**Isostrain**							
Exposed	745,818	329	4.41	156	2.09	56	0.75
Non-exposed	7,091,525	2,659	3.75	1,295	1.83	457	0.64
**Quadrants by Karasek**							
Active job	3,343,389	1,229	3.68	638	1.91	203	0.61
Low strain	1,249,108	362	2.90	169	1.35	64	0.51
Passive job	2,332,901	995	4.27	458	1.96	173	0.74
High strain	911,945	402	4.41	186	2.04	73	0.80
**CUMULATIVE EXPOSURE**							
**Job strain**							
Exposed	1,064,542	322	3.02	148	1.39	53	0.50
Non-exposed	9,258,496	2,666	2.88	1303	1.41	460	0.50
**Isostrain**							
Exposed	909,129	292	3.21	134	1.47	47	0.52
Non-exposed	9,413,909	2,696	2.86	1317	1.40	466	0.50
**Quadrants by Karasek**							
Active job	3,752,221	1,268	3.38	648	1.73	211	0.56
Low strain	2,500,201	464	1.86	224	0.90	85	0.34
Passive job	3,006,074	934	3.11	431	1.43	164	0.55
High strain	1,064,542	322	3.02	148	1.39	53	0.50
**RECENCY-WEIGHTED CUMULATIVE EXPOSURE**							
**Job strain**							
Exposed	1,054,827	337	3.19	156	1.48	62	0.59
Non-exposed	8,881,664	2,651	2.98	1295	1.46	451	0.51
**Isostrain**							
Exposed	880,169	289	3.28	135	1.53	49	0.56
Non-exposed	9,056,322	2,699	2.98	1316	1.45	464	0.51
**Quadrants by Karasek**							
Active job	3,941,509	1,269	3.22	655	1.66	210	0.53
Low strain	2,095,063	439	2.10	212	1.01	72	0.34
Passive job	2,845,092	943	3.31	428	1.50	169	0.59
High strain	1,054,827	337	3.19	156	1.48	62	0.59

Table S3. Person-years, number of cases, and cases per 10,000 person-years for the study of mortality until 2002 among men

	Person-years	Cardiovascular mortality		Mortality for ischemic heart diseases		Mortality for stroke	
		Number of cases	Cases per 10,000 person-years	Number of cases	Cases per 10,000 person-years	Number of cases	Cases per 10,000 person-years
**CUMULATIVE EXPOSURE**							
**Job strain**							
Exposed	1,407,544	2,425	17.23	946	6.72	469	3.33
Non-exposed	12,170,546	16,839	13.84	6,734	5.53	2,966	2.44
**Isostrain**							
Exposed	1,188,122	1,942	16.35	778	6.55	368	3.10
Non-exposed	12,389,967	17,322	13.98	6,902	5.57	3,067	2.48
**Quadrants by Karasek**							
Active job	5,012,322	7,258	14.48	3,122	6.23	1,217	2.43
Low strain	3,265,462	3,724	11.40	1,388	4.25	714	2.19
Passive job	3,892,761	5,857	15.05	2,224	5.71	1,035	2.66
High strain	1,407,544	2,425	17.23	946	6.72	469	3.33
**RECENCY-WEIGHTED CUMULATIVE EXPOSURE**							
**Job strain**							
Exposed	1,193,521	911	7.63	399	3.34	182	1.52
Non-exposed	9,971,541	6,389	6.41	2,877	2.89	1,128	1.13
**Isostrain**							
Exposed	988,946	722	7.30	322	3.26	134	1.35
Non-exposed	10,176,116	6,578	6.46	2,954	2.90	1,176	1.16
**Quadrants by Karasek**							
Active job	4,431,910	2,851	6.43	1,399	3.16	464	1.05
Low strain	2,369,125	1,320	5.57	549	2.32	253	1.07
Passive job	3,170,506	2,218	7.00	929	2.93	411	1.30
High strain	1,193,521	911	7.63	399	3.34	182	1.52

Table S4. Person-years, number of cases, and cases per 10,000 person-years for the study of on-the-job mortality among women

	Person-years	Cardiovascular mortality		Mortality for ischemic heart diseases		Mortality for stroke	
		Number of cases	Cases per 10,000 person-years	Number of cases	Cases per 10,000 person-years	Number of cases	Cases per 10,000 person-years
**CURRENT EXPOSURE**							
**Job strain**							
Exposed	1,551,634	143	0.92	26	0.17	60	0.39
Non-exposed	4,362,017	331	0.76	74	0.17	116	0.27
**Isostrain**							
Exposed	1,548,664	143	0.92	26	0.17	60	0.39
Non-exposed	4,364,988	331	0.76	74	0.17	116	0.27
**Quadrants by Karasek**							
Active job	2,449,155	151	0.62	33	0.13	54	0.22
Low strain	695,039	31	0.45	4	0.06	12	0.17
Passive job	1,217,822	149	1.22	37	0.30	50	0.41
High strain	1,551,634	143	0.92	26	0.17	60	0.39
**CUMULATIVE EXPOSURE**							
**Job strain**							
Exposed	2,951,312	184	0.62	36	0.12	71	0.24
Non-exposed	5,438,577	290	0.53	64	0.12	105	0.19
**Isostrain**							
Exposed	2,800,051	180	0.64	36	0.13	70	0.25
Non-exposed	5,589,838	294	0.53	64	0.11	106	0.19
**Quadrants by Karasek**							
Active job	2,990,960	139	0.46	28	0.09	56	0.19
Low strain	704,690	19	0.27	3	0.04	7	0.10
Passive job	1,742,927	132	0.76	33	0.19	42	0.24
High strain	2,951,312	184	0.62	36	0.12	71	0.24
**RECENCY-WEIGHTED CUMULATIVE EXPOSURE**							
**Job strain**							
Exposed	2,316,460	168	0.73	31	0.13	68	0.29
Non-exposed	5,553,816	306	0.55	69	0.12	108	0.19
**Isostrain**							
Exposed	2,238,737	165	0.74	30	0.13	67	0.30
Non-exposed	5,631,539	309	0.55	70	0.12	109	0.19
**Quadrants by Karasek**							
Active job	3,082,042	144	0.47	31	0.10	52	0.17
Low strain	790,405	25	0.32	5	0.06	9	0.11
Passive job	1,681,369	137	0.81	33	0.20	47	0.28
High strain	2,316,460	168	0.73	31	0.13	68	0.29

Table S5. Person-years, number of cases, and cases per 10,000 person-years for the study of mortality until 2002 among women

	Person-years	Cardiovascular mortality		Mortality for ischemic heart diseases		Mortality for stroke	
		Number of cases	Cases per 10,000 person-years	Number of cases	Cases per 10,000 person-years	Number of cases	Cases per 10,000 person-years
**CUMULATIVE EXPOSURE**							
**Job strain**							
Exposed	3,987,431	2,136	5.36	582	1.46	547	1.37
Non-exposed	7,574,327	4,045	5.34	994	1.31	1,070	1.41
**Isostrain**							
Exposed	3,800,697	2,092	5.50	574	1.51	532	1.40
Non-exposed	7,761,061	4,089	5.27	1,002	1.29	1,085	1.40
**Quadrants by Karasek**							
Active job	3,968,536	1,506	3.79	364	0.92	397	1.00
Low strain	1,014,635	452	4.45	112	1.10	136	1.34
Passive job	2,591,156	2,087	8.05	518	2.00	537	2.07
High strain	3,987,431	2,136	5.36	582	1.46	547	1.37
**RECENCY-WEIGHTED CUMULATIVE EXPOSURE**							
**Job strain**							
Exposed	2,606,873	428	1.64	93	0.36	137	0.53
Non-exposed	6,403,404	939	1.47	214	0.33	315	0.49
**Isostrain**							
Exposed	2,522,472	419	1.66	92	0.36	133	0.53
Non-exposed	6,487,806	948	1.46	215	0.33	319	0.49
**Quadrants by Karasek**							
Active job	3,462,904	374	1.08	81	0.23	125	0.36
Low strain	932,628	112	1.20	25	0.27	38	0.41
Passive job	2,007,872	453	2.26	108	0.54	152	0.76
High strain	2,606,873	428	1.64	93	0.36	137	0.53

Table S6. Associations between current exposure and mortality for ischemic heart diseases among men and women

	MEN (N=798,547)	WOMEN (N=697,785)
	HR (95% CI)<br>(Ischemic heart disease deaths=1,451)	HR (95% CI)<br>(Ischemic heart disease deaths=100)
High psychological demands[1]	0.94 [0.82-1.09]	0.96 [0.61-1.51]
Low decision latitude[1]	**1.24 [1.09-1.40]**	**2.34 [1.32-4.15]**
Low social support[1]	**1.16 [1.03-1.32]**	1.43 [0.91-2.24]
High psychological demands[2]	1.01 [0.87-1.18]	1.08 [0.66-1.77]
Low decision latitude[2]	**1.27 [1.05-1.55]**	**2.37 [1.19-4.73]**
Low social support[2]	0.96 [0.79-1.18]	1.00 [0.58-1.73]
Job strain[1]	1.18 [1.00-1.40]	1.21 [0.71-2.08]
Isostrain[1]	1.17 [0.98-1.40]	1.22 [0.71-2.09]
**Quadrants by Karasek**[1]		
Active job (ref)	1	1
Low strain	0.99 [0.81-1.20]	0.44 [0.15-1.25]
Passive job	**1.22 [1.03-1.44]**	**2.23 [1.13-4.38]**
High strain	**1.24 [1.04-1.49]**	1.80 [0.92-3.51]

[1] Each exposure was studied separately

[2] Demands, latitude and support were studied simultaneously, i.e. adjusted for each other

High strain (high demands and low latitude), low strain (low demands and high latitude), passive job (low demands and low latitude), and active job, the reference group (high demands and high latitude)

All models were adjusted for calendar time, biomechanical, physical, chemical and biological exposures

Age was used as the time scale

Table S7. Associations between cumulative exposure and mortality for ischemic heart diseases among men and women

	MEN N=798,547		WOMEN N=697,785	
Follow-up	**On-the-job**	**Until 31/12/2002**	**On-the-job**	**Until 31/12/2002**
	HR (95% CI) (Ischemic heart disease deaths=1,451)	HR (95% CI) (Ischemic heart disease deaths=7,680)	HR (95% CI) (Ischemic heart disease deaths=100)	HR (95% CI) (Ischemic heart disease deaths=1,576)
High psychological demands[1]	1.04 [0.91-1.20]	0.98 [0.92-1.04]	0.83 [0.52-1.30]	0.91 [0.81-1.02]
Low decision latitude[1]	**1.30 [1.15-1.46]**	**1.17 [1.12-1.24]**	**1.73 [1.03-2.92]**	**1.33 [1.16-1.53]**
Low social support[1]	**1.22 [1.09-1.37]**	**1.08 [1.03-1.14]**	1.37 [0.85-2.21]	1.12 [0.99-1.26]
High psychological demands[2]	1.13 [0.98-1.31]	1.01 [0.95-1.08]	0.84 [0.51-1.38]	0.93 [0.82-1.05]
Low decision latitude[2]	**1.27 [1.11-1.46]**	**1.17 [1.11-1.24]**	1.54 [0.86-2.76]	**1.28 [1.10-1.49]**
Low social support[2]	1.11 [0.97-1.26]	1.01 [0.96-1.07]	1.24 [0.73-2.12]	1.06 [0.93-1.22]
Job strain[1]	**1.24 [1.04-1.48]**	**1.17 [1.09-1.26]**	1.04 [0.65-1.67]	1.09 [0.96-1.24]
Isostrain[1]	**1.25 [1.04-1.50]**	**1.18 [1.09-1.27]**	1.12 [0.70-1.80]	1.10 [0.97-1.25]
**Quadrants by Karasek**[1]				
Active job (ref)	1	1	1	1
Low strain	0.84 [0.70-1.00]	1.01 [0.94-1.09]	0.54 [0.16-1.80]	1.09 [0.88-1.36]
Passive job	1.17 [0.99-1.38]	**1.16 [1.08-1.24]**	**1.89 [1.00-3.55]**	**1.40 [1.18-1.65]**
High strain	**1.26 [1.04-1.52]**	**1.22 [1.13-1.32]**	1.41 [0.78-2.54]	**1.34 [1.14-1.58]**

[1] Each exposure was studied separately

[2] Demands, latitude and support were studied simultaneously, i.e. adjusted for each other

High strain (high demands and low latitude), low strain (low demands and high latitude), passive job (low demands and low latitude), and active job, the reference group (high demands and high latitude)

All models were adjusted for calendar time, biomechanical, physical, chemical and biological exposures

Age was used as the time scale

Table S8. Associations between recency-weighted cumulative exposure and mortality for ischemic heart diseases among men and women

	MEN N=798,547		WOMEN N=697,785	
Follow-up	**On-the-job**	**Until 31/12/2002**	**On-the-job**	**Until 31/12/2002**
	HR (95% CI) (Ischemic heart disease deaths=1,451)	HR (95% CI) (Ischemic heart disease deaths=7,680)	HR (95% CI) (Ischemic heart disease deaths=100)	HR (95% CI) (Ischemic heart disease deaths=1,576)
High psychological demands[1]	0.99 [0.86-1.14]	0.98 [0.90-1.08]	0.97 [0.61-1.52]	0.96 [0.74-1.25]
Low decision latitude[1]	**1.23 [1.09-1.38]**	**1.17 [1.08-1.27]**	1.63 [0.94-2.82]	1.32 [0.96-1.81]
Low social support[1]	**1.23 [1.10-1.39]**	**1.12 [1.04-1.21]**	**1.70 [1.06-2.73]**	1.11 [0.86-1.44]
High psychological demands[2]	1.06 [0.92-1.23]	1.03 [0.93-1.13]	0.92 [0.56-1.51]	1.00 [0.76-1.33]
Low decision latitude[2]	1.14 [0.98-1.32]	**1.16 [1.05-1.28]**	1.26 [0.67-2.39]	1.31 [0.90-1.90]
Low social support[2]	**1.16 [1.00-1.34]**	1.03 [0.94-1.14]	1.58 [0.92-2.72]	1.01 [0.75-1.36]
Job strain[1]	1.16 [0.97-1.38]	**1.20 [1.08-1.35]**	1.15 [0.70-1.89]	1.14 [0.86-1.52]
Isostrain[1]	1.14 [0.95-1.37]	**1.16 [1.03-1.31]**	1.13 [0.68-1.87]	1.16 [0.87-1.55]
**Quadrants by Karasek**[1]				
Active job (ref)	1	1	1	1
Low strain	0.92 [0.77-1.11]	1.02 [0.91-1.14]	0.66 [0.25-1.73]	1.04 [0.65-1.64]
Passive job	1.18 [1.00-1.39]	**1.15 [1.03-1.28]**	1.58 [0.82-3.06]	1.31 [0.90-1.92]
High strain	1.20 [0.99-1.45]	**1.25 [1.11-1.41]**	1.43 [0.76-2.69]	1.35 [0.92-1.97]

[1] Each exposure was studied separately

[2] Demands, latitude and support were studied simultaneously, i.e. adjusted for each other

High strain (high demands and low latitude), low strain (low demands and high latitude), passive job (low demands and low latitude), and active job, the reference group (high demands and high latitude)

All models were adjusted for calendar time, biomechanical, physical, chemical and biological exposures

Age was used as the time scale

Table S9. Associations between current exposure and mortality for stroke among men and women

	MEN (N=798,547)	WOMEN (N=697,785)
	HR (95% CI) (Stroke deaths=513)	HR (95% CI) (Stroke deaths=176)
High psychological demands[1]	0.90 [0.71-1.14]	1.08 [0.76-1.53]
Low decision latitude[1]	**1.36 [1.12-1.67]**	1.55 [0.99-2.41]
Low social support[1]	1.16 [0.94-1.42]	**1.43 [1.00-2.03]**
High psychological demands[2]	0.96 [0.75-1.24]	1.11 [0.76-1.61]
Low decision latitude[2]	**1.63 [1.19-2.25]**	1.38 [0.79-2.39]
Low social support[2]	0.78 [0.56-1.08]	1.24 [0.80-1.90]
Job strain[1]	**1.33 [1.01-1.75]**	1.34 [0.90-2.00]
Isostrain[1]	1.19 [0.89-1.60]	1.35 [0.91-2.01]
**Quadrants by Karasek**[1]		
Active job (ref)	1	1
Low strain	1.08 [0.78-1.49]	0.84 [0.44-1.60]
Passive job	**1.37 [1.04-1.80]**	1.38 [0.82-2.31]
High strain	**1.47 [1.09-1.98]**	1.59 [0.95-2.67]

[1] Each exposure was studied separately
[2] Demands, latitude and support were studied simultaneously, i.e. adjusted for each other
High strain (high demands and low latitude), low strain (low demands and high latitude), passive job (low demands and low latitude), and active job, the reference group (high demands and high latitude)
All models were adjusted for calendar time, biomechanical, physical, chemical and biological exposures
Age was used as the time scale

Table S10. Associations between cumulative exposure and mortality for stroke among men and women

	MEN N=798,547		WOMEN N=697,785	
Follow-up	**On-the-job**	**Until 31/12/2002**	**On-the-job**	**Until 31/12/2002**
	HR (95% CI) (Stroke deaths=513)	HR (95% CI) (Stroke deaths=3,435)	HR (95% CI) (Stroke deaths=176)	HR (95% CI) (Stroke deaths=1,617)
High psychological demands[1]	0.97 [0.77-1.23]	0.93 [0.85-1.01]	1.19 [0.83-1.72]	**0.86 [0.76-0.96]**
Low decision latitude[1]	**1.36 [1.11-1.65]**	**1.18 [1.09-1.27]**	1.36 [0.92-2.01]	**1.33 [1.16-1.52]**
Low social support[1]	**1.27 [1.05-1.55]**	1.04 [0.97-1.12]	**2.22 [1.47-3.36]**	1.08 [0.96-1.21]
High psychological demands[2]	1.06 [0.83-1.35]	0.96 [0.88-1.05]	1.06 [0.72-1.58]	0.88 [0.78-1.00]
Low decision latitude[2]	**1.29 [1.03-1.62]**	**1.20 [1.10-1.30]**	1.03 [0.67-1.59]	**1.28 [1.10-1.48]**
Low social support[2]	1.14 [0.92-1.43]	0.96 [0.88-1.04]	**2.18 [1.39-3.41]**	1.03 [0.91-1.18]
Job strain[1]	1.23 [0.91-1.66]	**1.29 [1.16-1.44]**	1.34 [0.94-1.90]	1.10 [0.97-1.24]
Isostrain[1]	1.22 [0.90-1.67]	**1.25 [1.11-1.40]**	1.39 [0.98-1.99]	1.08 [0.95-1.23]
**Quadrants by Karasek[1]**				
Active job (ref)	1	1	1	1
Low strain	0.90 [0.67-1.22]	**1.19 [1.06-1.32]**	0.68 [0.31-1.50]	**1.27 [1.04-1.56]**
Passive job	1.28 [0.97-1.69]	**1.22 [1.10-1.36]**	1.13 [0.69-1.84]	**1.46 [1.24-1.72]**
High strain	1.30 [0.94-1.79]	**1.42 [1.26-1.59]**	1.38 [0.89-2.12]	**1.39 [1.18-1.63]**

[1] Each exposure was studied separately
[2] Demands, latitude and support were studied simultaneously, i.e. adjusted for each other
High strain (high demands and low latitude), low strain (low demands and high latitude), passive job (low demands and low latitude), and active job, the reference group (high demands and high latitude)
All models were adjusted for calendar time, biomechanical, physical, chemical and biological exposures
Age was used as the time scale

Table S11. Associations between recency-weighted cumulative exposure and mortality for stroke among men and women

	MEN N=798,547		WOMEN N=697,785	
Follow-up	**On-the-job**	**Until 31/12/2002**	**On-the-job**	**Until 31/12/2002**
	HR (95% CI) (Stroke deaths=513)	HR (95% CI) (Stroke deaths=3,435)	HR (95% CI) (Stroke deaths=176)	HR (95% CI) (Stroke deaths=1,617)
High psychological demands[1]	0.97 [0.77-1.22]	0.89 [0.77-1.03]	1.15 [0.81-1.64]	**0.78 [0.63-0.97]**
Low decision latitude[1]	**1.46 [1.20-1.78]**	**1.30 [1.15-1.47]**	**1.81 [1.19-2.75]**	**1.59 [1.23-2.06]**
Low social support[1]	**1.29 [1.06-1.58]**	1.07 [0.95-1.21]	**1.65 [1.15-2.36]**	1.20 [0.97-1.48]
High psychological demands[2]	1.09 [0.86-1.40]	0.94 [0.81-1.09]	1.19 [0.82-1.74]	0.81 [0.65-1.02]
Low decision latitude[2]	**1.43 [1.11-1.84]**	1.40 [1.19-1.64]	1.61 [0.99-2.61]	**1.46 [1.08-1.98]**
Low social support[2]	1.07 [0.83-1.37]	0.87 [0.74-1.02]	1.34 [0.88-2.04]	1.08 [0.85-1.38]
Job strain[1]	**1.34 [1.01-1.77]**	**1.41 [1.19-1.67]**	**1.59 [1.11-2.28]**	1.08 [0.85-1.37]
Isostrain[1]	1.19 [0.88-1.62]	**1.22 [1.01-1.47]**	**1.62 [1.12-2.33]**	1.06 [0.83-1.35]
**Quadrants by Karasek**[1]				
Active job (ref)	1	1	1	1
Low strain	0.89 [0.65-1.22]	**1.26 [1.05-1.51]**	0.80 [0.39-1.65]	1.24 [0.85-1.79]
Passive job	**1.36 [1.03-1.80]**	**1.37 [1.15-1.63]**	1.49 [0.90-2.47]	**1.85 [1.35-2.52]**
High strain	**1.44 [1.06-1.95]**	**1.62 [1.35-1.96]**	**1.92 [1.21-3.04]**	**1.56 [1.15-2.12]**

[1] Each exposure was studied separately

[2] Demands, latitude and support were studied simultaneously, i.e. adjusted for each other

High strain (high demands and low latitude), low strain (low demands and high latitude), passive job (low demands and low latitude), and active job, the reference group (high demands and high latitude)

All models were adjusted for calendar time, biomechanical, physical, chemical and biological exposures

Age was used as the time scale

Table S12. Associations between current exposure and cardiovascular mortality among men and women (with additional adjustment for occupation)

	MEN (N=798,547)	WOMEN (N=697,785)
	HR (95% CI)<br>(Cardiovascular deaths=2,988)	HR (95% CI)<br>(Cardiovascular deaths=474)
High psychological demands[a]	0.96 [0.86-1.06]	1.17 [0.93-1.46]
Low decision latitude[a]	**1.28 [1.16-1.41]**	**1.45 [1.05-2.02]**
Low social support[a]	**1.13 [1.03-1.25]**	**1.51 [1.12-2.05]**
High psychological demands[b]	0.99 [0.89-1.11]	1.02 [0.78-1.32]
Low decision latitude[b]	**1.38 [1.20-1.58]**	1.33 [0.95-1.87]
Low social support[b]	0.90 [0.78-1.03]	1.41 [0.99-2.00]
Job strain[a]	**1.16 [1.01-1.34]**	1.18 [0.92-1.51]
Isostrain[a]	1.09 [0.95-1.25]	1.18 [0.92-1.52]
**Quadrants by Karasek**[a]		
Active job (ref)	1	1
Low strain	0.99 [0.85-1.16]	0.69 [0.46-1.04]
Passive job	**1.28 [1.11-1.46]**	1.22 [0.82-1.82]
High strain	**1.28 [1.08-1.51]**	1.29 [0.88-1.87]

[a] Each exposure was studied separately
[b] Demands, latitude and support were studied simultaneously, i.e. adjusted for each other
High strain (high demands and low latitude), low strain (low demands and high latitude), passive job (low demands and low latitude), and active job, the reference group (high demands and high latitude)
All models were adjusted for calendar time, biomechanical, physical, chemical and biological exposures, and occupation
Age was used as the time scale

Table S13. Associations between cumulative exposure and cardiovascular mortality among men and women (with additional adjustment for occupation)

	MEN N=798,547		WOMEN N=697,785	
Follow-up	**On-the-job**	**Until 31/12/2002**	**On-the-job**	**Until 31/12/2002**
	HR (95% CI) (Cardiovascular deaths=2,988)	HR (95% CI) (Cardiovascular deaths=19,264)	HR (95% CI) (Cardiovascular deaths=474)	HR (95% CI) (Cardiovascular deaths=6,181)
High psychological demands[a]	1.08 [0.98-1.20]	0.98 [0.94-1.02]	1.11 [0.88-1.38]	0.92 [0.87-0.98]
Low decision latitude[a]	**1.36 [1.24-1.48]**	**1.13 [1.10-1.17]**	**1.41 [1.08-1.85]**	**1.23 [1.14-1.33]**
Low social support[a]	**1.17 [1.08-1.27]**	**1.04 [1.01-1.08]**	**1.54 [1.21-1.97]**	1.01 [0.95-1.07]
High psychological demands[b]	**1.15 [1.03-1.28]**	1.00 [0.96-1.04]	1.01 [0.79-1.28]	0.94 [0.88-1.00]
Low decision latitude[b]	**1.36 [1.23-1.50]**	**1.14 [1.10-1.18]**	1.26 [0.96-1.67]	**1.23 [1.13-1.33]**
Low social support[b]	1.04 [0.95-1.14]	0.99 [0.95-1.02]	**1.46 [1.13-1.90]**	1.00 [0.93-1.07]
Job strain[a]	**1.23 [1.08-1.40]**	**1.11 [1.06-1.17]**	1.20 [0.96-1.49]	1.02 [0.96-1.09]
Isostrain[a]	**1.24 [1.08-1.41]**	**1.09 [1.04-1.15]**	1.23 [0.99-1.53]	1.01 [0.95-1.08]
**Quadrants by Karasek[a]**				
Active job (ref)	1	1	1	1
Low strain	0.80 [0.70-0.91]	1.02 [0.97-1.07]	0.67 [0.41-1.09]	1.03 [0.92-1.15]
Passive job	**1.18 [1.04-1.34]**	**1.14 [1.08-1.19]**	1.26 [0.90-1.76]	**1.30 [1.18-1.43]**
High strain	**1.23 [1.07-1.43]**	**1.16 [1.10-1.22]**	1.32 [0.98-1.77]	**1.20 [1.10-1.32]**

[a] Each exposure was studied separately
[b] Demands, latitude and support were studied simultaneously, i.e. adjusted for each other
High strain (high demands and low latitude), low strain (low demands and high latitude), passive job (low demands and low latitude), and active job, the reference group (high demands and high latitude)
All models were adjusted for calendar time, biomechanical, physical, chemical and biological exposures, and occupation
Age was used as the time scale

Table S14. Associations between recency-weighted cumulative exposure and cardiovascular mortality among men and women (with additional adjustment for occupation)

	MEN N=798,547		WOMEN N=697,785	
Follow-up	**On-the-job**	**Until 31/12/2002**	**On-the-job**	**Until 31/12/2002**
	HR (95% CI) (Cardiovascular deaths=2,988)	HR (95% CI) (Cardiovascular deaths=19,264)	HR (95% CI) (Cardiovascular deaths=474)	HR (95% CI) (Cardiovascular deaths=6,181)
High psychological demands[a]	1.01 [0.91-1.12]	1.00 [0.94-1.06]	**1.26 [1.00-1.57]**	0.95 [0.84-1.08]
Low decision latitude[a]	**1.31 [1.19-1.43]**	**1.13 [1.08-1.20]**	**1.52 [1.12-2.06]**	**1.31 [1.12-1.52]**
Low social support[a]	**1.23 [1.12-1.34]**	1.02 [0.97-1.08]	**1.80 [1.38-2.34]**	1.08 [0.95-1.22]
High psychological demands[b]	1.05 [0.95-1.17]	1.03 [0.96-1.09]	1.08 [0.85-1.38]	0.98 [0.86-1.12]
Low decision latitude[b]	**1.25 [1.13-1.39]**	**1.19 [1.12-1.27]**	1.33 [0.97-1.81]	**1.31 [1.10-1.56]**
Low social support[b]	1.10 [0.99-1.22]	0.93 [0.87-0.99]	**1.65 [1.24-2.20]**	0.99 [0.86-1.14]
Job strain[a]	1.14 [0.99-1.31]	**1.15 [1.07-1.24]**	**1.35 [1.08-1.70]**	1.10 [0.96-1.26]
Isostrain[a]	1.10 [0.96-1.26]	1.07 [0.99-1.16]	**1.37 [1.09-1.72]**	1.10 [0.96-1.26]
**Quadrants by Karasek[a]**				
Active job (ref)	1	1	1	1
Low strain	0.88 [0.76-1.01]	1.00 [0.92-1.08]	0.70 [0.45-1.09]	1.03 [0.82-1.27]
Passive job	**1.22 [1.07-1.40]**	**1.11 [1.03-1.19]**	1.20 [0.83-1.74]	**1.33 [1.10-1.60]**
High strain	**1.18 [1.01-1.39]**	**1.19 [1.09-1.29]**	**1.46 [1.04-2.04]**	**1.31 [1.09-1.56]**

[a] Each exposure was studied separately
[b] Demands, latitude and support were studied simultaneously, i.e. adjusted for each other
High strain (high demands and low latitude), low strain (low demands and high latitude), passive job (low demands and low latitude), and active job, the reference group (high demands and high latitude)
All models were adjusted for calendar time, biomechanical, physical, chemical and biological exposures, and occupation
Age was used as the time scale

Figure S1. Kaplan-Meier survival curves according to current exposure to job strain among men and women (adjusted for calendar time and other occupational exposures)

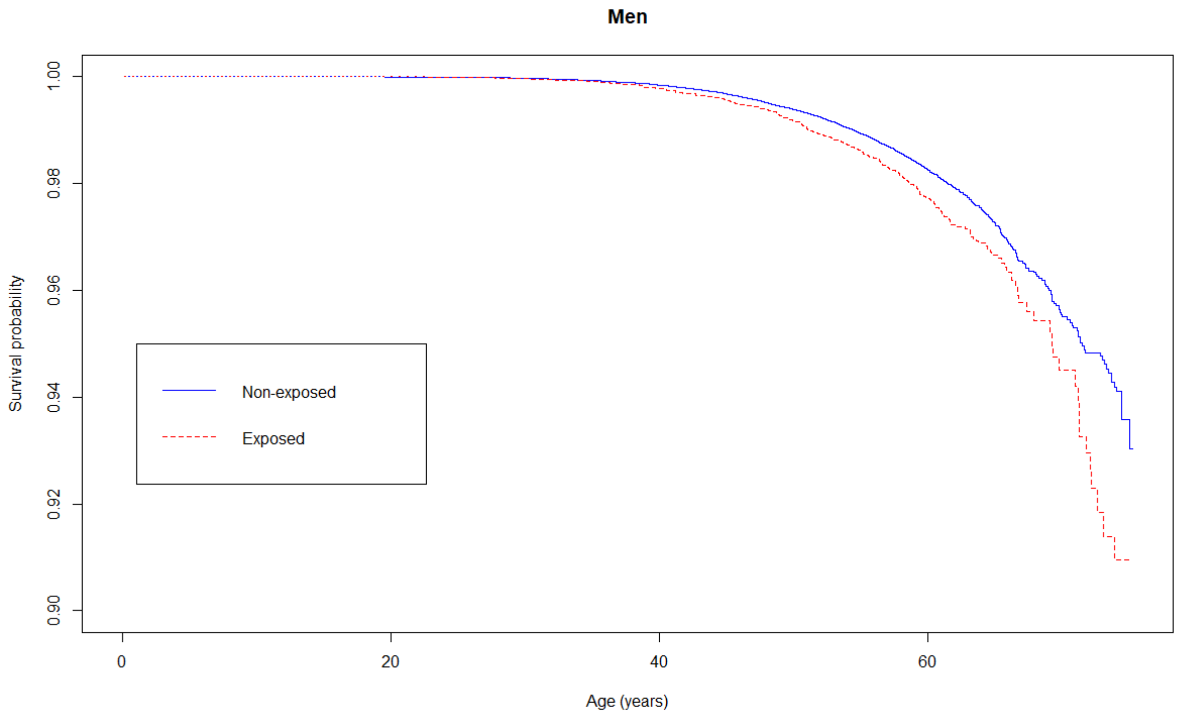

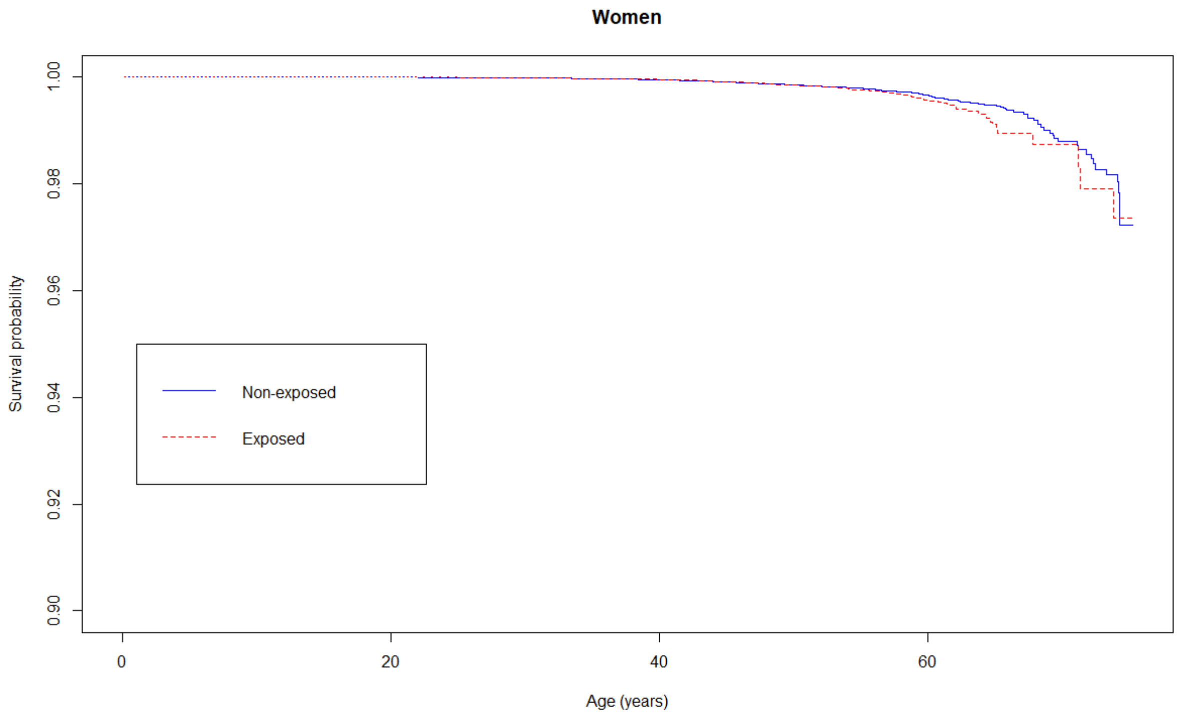

Figure S2. Kaplan-Meier survival curves according to current exposure to iso-strain among men and women (adjusted for calendar time and other occupational exposures)

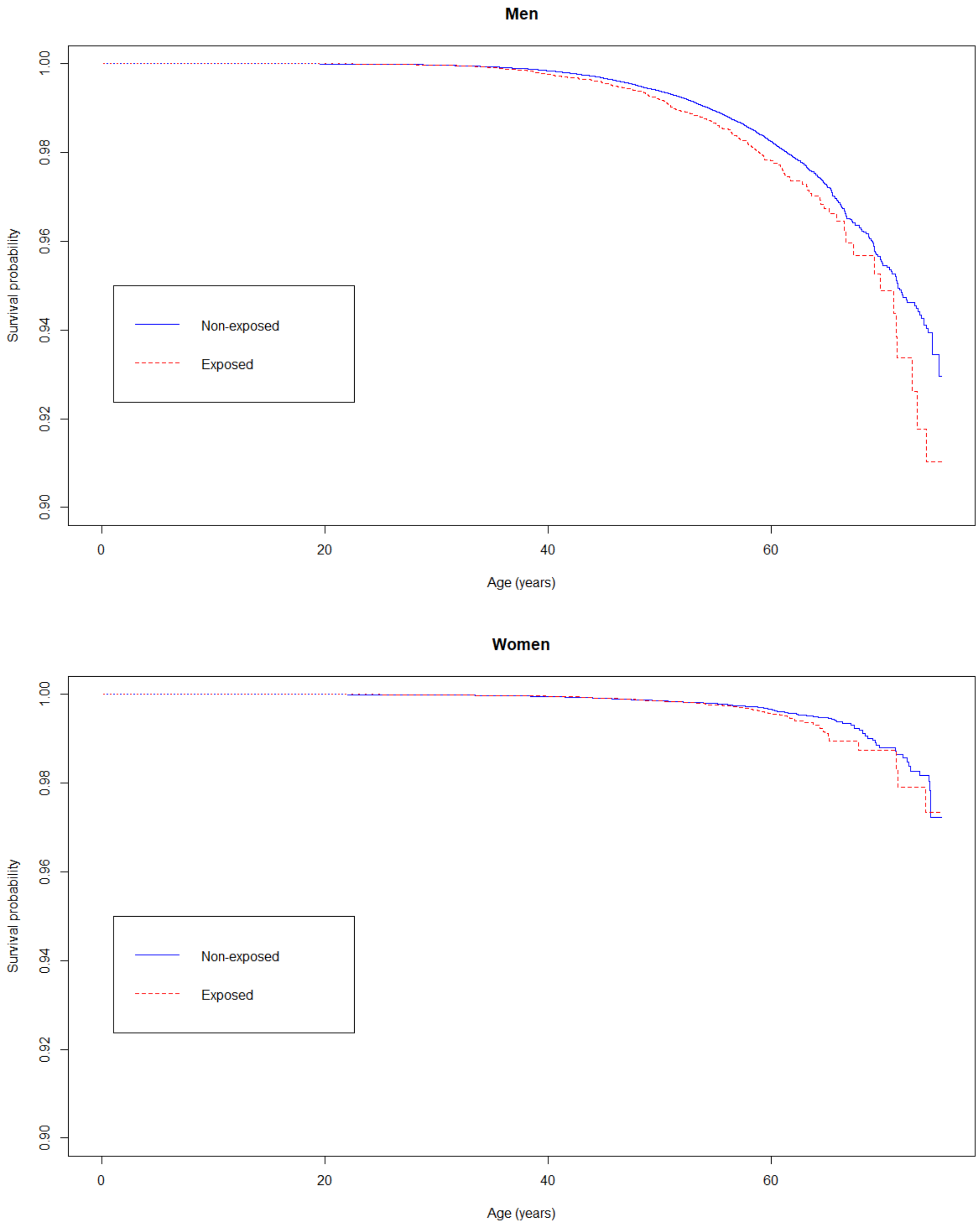

Figure S3. Kaplan-Meier survival curves according to Karasek's job situations among men and women (adjusted for calendar time and other occupational exposures)

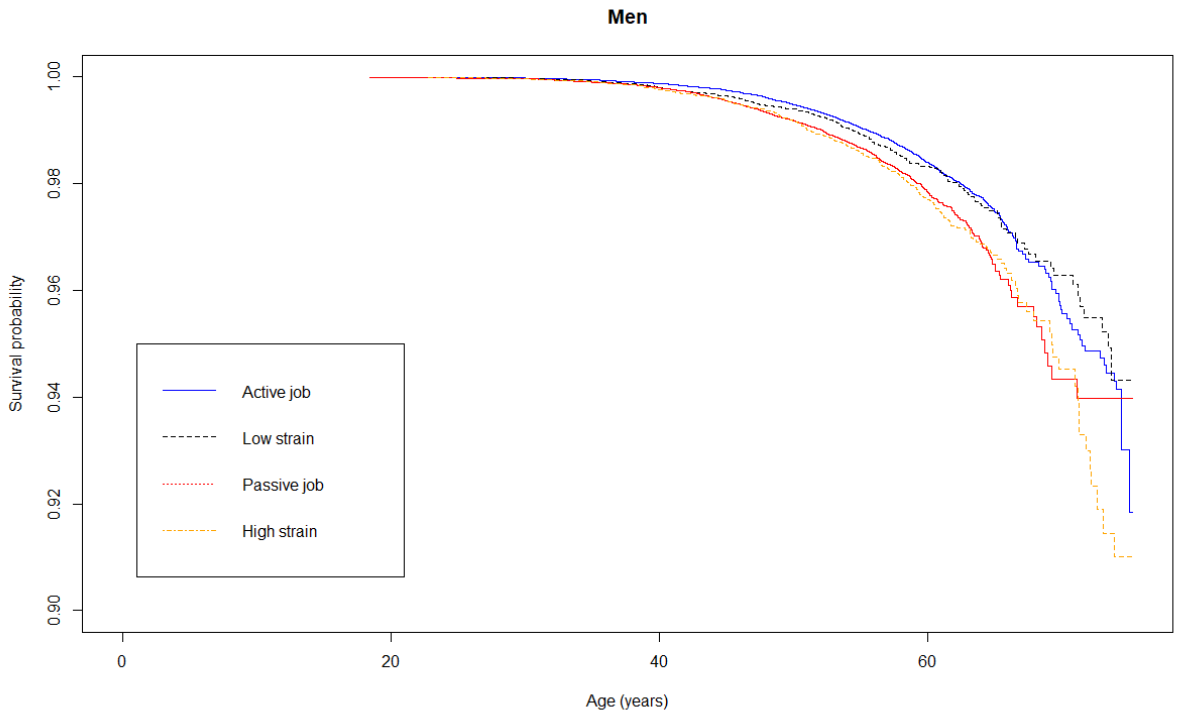

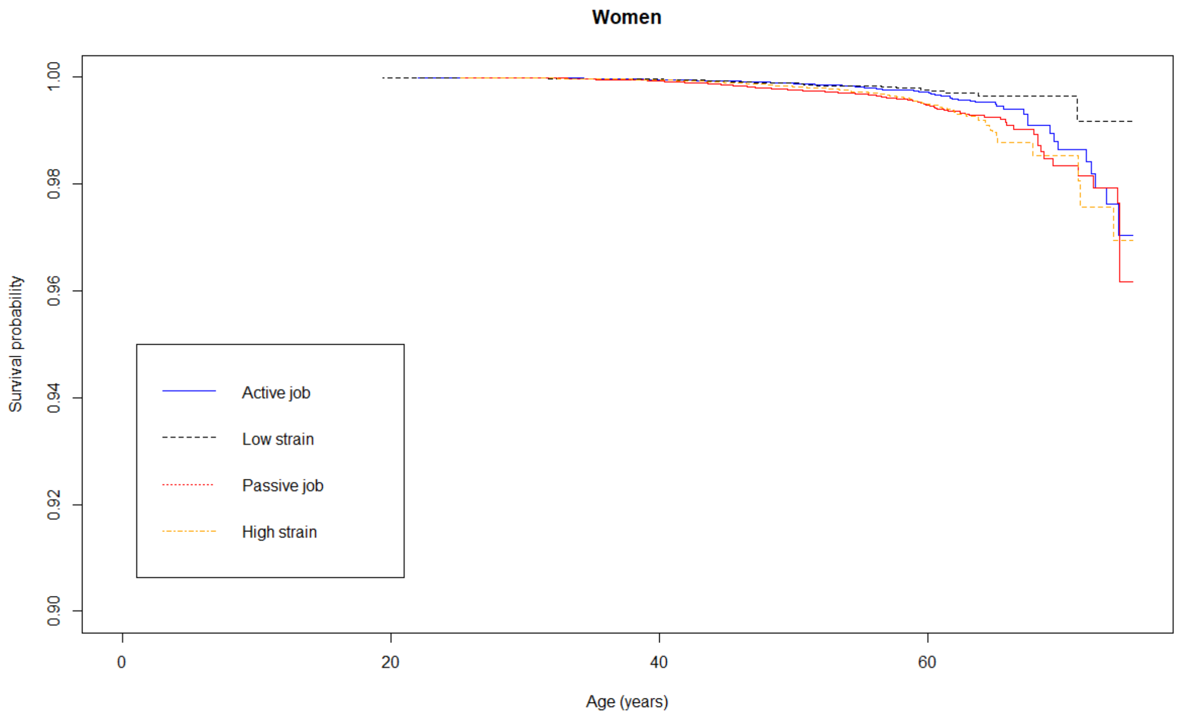

Supplement: Supplementary material [file SJWEH-46-542-S001.pdf]
